# pygenstrat: a Python package for EIGENSTRAT data processing

**DOI:** 10.1093/bioadv/vbag022

**Published:** 2026-01-23

**Authors:** Dilek Koptekin

**Affiliations:** Department of Computational Biology, University of Lausanne, Lausanne 1015, Switzerland; Swiss Institute of Bioinformatics, Lausanne 1015, Switzerland

## Abstract

**Motivation:**

Ancient DNA studies rely heavily on the EIGENSTRAT genotype format (.geno, .ind, .snp) for standard population genetic analyses including PCA, f-statistics, and qpWave/qpAdm. However, there is limited software available for processing EIGENSTRAT format data. ***pygenstrat***, a Python package, is presented here, providing a command-line interface for comprehensive EIGENSTRAT data processing with extensive filtering, subsetting, and conversion options. *pygenstrat* implements memory-efficient, chunked processing algorithms for handling large ancient DNA datasets with low memory usage. It supports comprehensive operations, including updating individual and SNP files, subsetting datasets by selecting individuals or SNPs, filtering by minor allele frequency and missingness, pseudo-haploidisation, allele polarization, as well as conversion between EIGENSTRAT (text) and ANCESTRYMAP (binary) formats. Its modular architecture and Python implementation enable rapid integration with custom pipelines and future extensions.

**Results:**

Benchmarking on the Allen Ancient DNA Resource (v 62.0) shows 2×–15× speedups and 90%–95% memory reduction compared to *convertf*, while producing equivalent outputs for standard operations. These improvements reduce turnaround time in ancient DNA workflows and facilitate reproducible processing.

**Availability and implementation:**

*pygenstrat* is open-source, available at https://github.com/dkoptekin/pygenstrat.

## 1 Introduction

Over the last decade, EIGENSTRAT file formats (.geno, .snp, .ind) have become the standard for population genetic analyses in ancient DNA analysis. These files are required as an input for standard population genetic analyses in ancient DNA studies, including PCA, f-statistics, and modelling with the qpWave/qpAdm framework using EIGENSOFT (Patterson, Price and Reich 2006) or ADMIXTOOLS (Patterson *et al.* 2012). The EIGENSTRAT format consists of three files: a genotype file (.geno) containing one line per SNP with genotype calls for each individual; a SNP file (.snp) with SNP information including chromosome, genetic position, physical position and alleles; and an individual file (.ind) listing sample ID, sex, and population label for each individual. The main tool for manipulating EIGENSTRAT data is *convertf*, implemented in both ADMIXTOOLS and EIGENSOFT software, which are designed for format conversion and basic subsetting of EIGENSTRAT files.

The field’s dependence on *convertf*, has created significant computational bottlenecks when processing the large-scale genomic datasets. *convertf* supports conversion while offering a few filtering options, such as keep individual and remove snps. Since *convertf* has limited filtering options, researchers often need to use multi-step workflows and external tools like PLINK (Chang *et al.* 2015) for basic data manipulation. For example, ancient DNA studies usually require filtering samples or SNPs based on the number of genotyped SNPs per sample or the frequency of genotyping per SNP. To do this, researchers need to calculate missing rates either per individual or per SNP. Currently, this involves converting EIGENSTRAT files to PLINK format, running missingness calculations and subsetting in PLINK, and then converting the data back to EIGENSTRAT, adding complexity and extra steps to the workflow. This fragmented process slows down analysis, increases the risk of errors, and makes workflows harder to reproduce.

Alternative tools have been developed to address some of these limitations. EigenStratDatabaseTools (Lamnidis 2022) provides three scripts for sample selection, renaming SNPs, and calculating the number of SNPs per individual, but does not offer integrated filtering or format conversion capabilities. The Poseidon framework (Schmid *et al*. 2024) provides a data management system with its command-line tool ‘trident’, supporting package-based data organization with structured metadata, format conversion, dataset merging, and validation workflows. However, Poseidon requires data to be organized in a specific package format with metadata files, which may not be practical for existing datasets or workflows. While these tools address specific needs, they do not provide extensive filtering and statistical functionality, leaving similar workflow complexities as with convertf.

**Figure 1:**
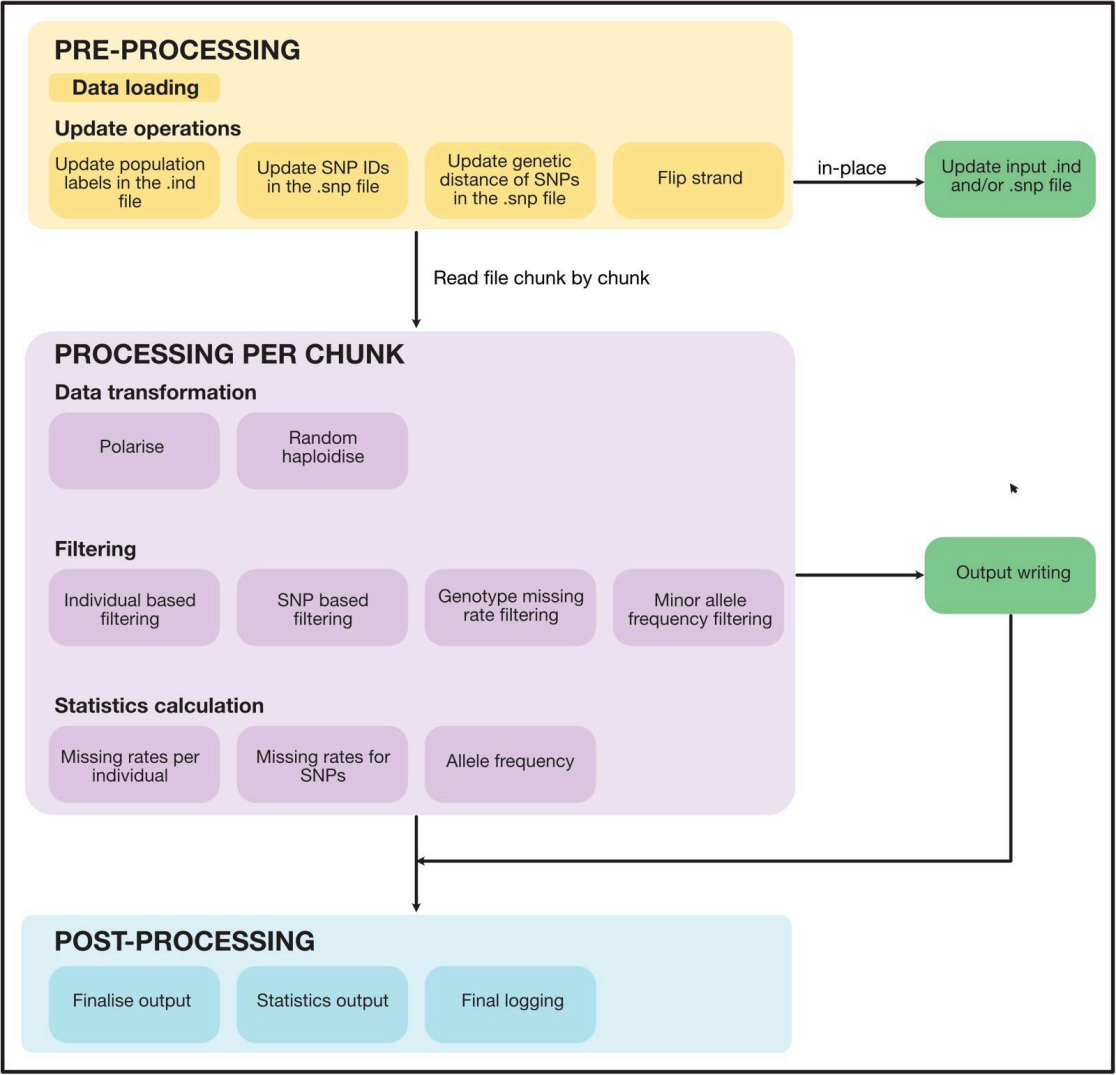
*pygenstrat* chunk-based processing workflow and architecture.

*pygenstrat*, presented here, is a Python package that provides comprehensive EIGENSTRAT file processing capabilities with substantial performance improvements and extended functionality, specifically designed for ancient DNA research workflows which can considerably speed up ancient DNA research workflows where these files are commonly used (Fig. 1).

## 2 Methods

*pygenstrat* provides EIGENSTRAT data processing through a command-line interface, designed around memory-efficient chunked processing algorithms that enable analysis of large ancient DNA datasets while maintaining consistently low memory usage. Unlike *convertf*, which load entire datasets into memory, *pygenstrat* processes genotype data in configurable, user-defined chunks (default 1000 SNPs), allowing the analysis of datasets that exceed available system memory and ensuring stable performance across varying dataset sizes.

*pygenstrat* accepts EIGENSTRAT format inputs (.geno, .snp, .ind) and automatically detects whether files are in text (EIGENSTRAT) or binary (ANCESTRYMAP) format. As shown in Fig. 1, it supports two distinct operation modes that determine how data is processed and where outputs are written.

**Update-only mode:** allows for efficient modification of metadata files (.ind and/or .snp files) without processing the large genotype data (.geno file). When using only metadata update options (‘- -update-ind’, ‘- -update-snp’, ‘- -genetic-map’, or ‘- -flip-strand’), *pygenstrat* modifies these files directly in their original location (in-place) rather than generating new output files. Before modifying any file, the original file is automatically preserved with a .backup extension. This mode is optimized for quick metadata updates without data processing overhead.

*Population label updates (‘- -update-ind’)* reassign individual population labels by matching sample IDs using a two-column mapping file.

*SNP identifier updates (‘- -update-snp’)* replace SNP identifiers (SNP IDs) using a two-column mapping file, enabling standardization across datasets.

*Genetic map integration (‘- -genetic-map’)* re-calculates genetic map positions (genetic distance) via per-chromosome interpolation with units controlled by –map-unit (cM or M).

*Flip-strand (‘- -flip-strand’)* updates allele orientation by flipping alleles to their complement version using a file containing SNP IDs.

If update options are combined with ‘- -out’ or any flags in processing mode, the updates are written only to the designated outputs, leaving original inputs unchanged.

*Processing mode:* When filtering, statistics, conversion, or other processing options are specified, all operations are processed in configurable chunks with results written chunk-by-chunk to new output files. This mode handles the full data processing pipeline while maintaining low memory usage regardless of dataset size.

*Individual and population filtering* allow users to include and exclude specific individuals or populations that listed in simple text files using keep (‘- -keep-indv’, ‘- -keep-pop’) or remove (‘- -remove-indv’, ‘- -remove-pop’) operations. Filtering is applied once globally to create individual masks used across all chunks.

*SNP selection and filtering* can be done using three different approaches: (i) SNP ID based lists (‘- -keep-snps’, ‘- -remove-snps’) for panel-based or custom selection, (ii) Genomic region-based selections (‘- -keep-region’, ‘- -remove-region’) using either an interval file or direct specifications like ‘1:1 000 000–2 000 000’ with interval-tree matching using *intervaltree* python package (https://pypi.org/project/intervaltree) that enable fast lookup of overlapping genomic regions, and (iii) Chromosome-based selection (- -keep-chr, - -remove-chr) supporting comma-separated sets and ranges (e.g. ‘1,2,5–6,22’) with or without chr prefix.

*Quality control filtering* removes SNPs based on (i) missing data thresholds (‘- -geno’), ensuring that only well-genotyped SNPs are used, and/or (ii) minor allele frequency thresholds (‘- -min-maf’, ‘- -max-maf’), which remove rare variants or possible artefacts, thereby improving downstream analysis robustness. *pygenstrat* detects sex chromosomes based on user-defined identifiers (configurable via- -sex-chr, default: 23,24) and applies appropriate statistical calculations. For Y chromosome SNPs, statistics are calculated for only male individuals (sex=M in the .ind file), since females (sex=F in the .ind file) should not carry Y chromosome variants. For X chromosome SNPs, statistics include all individuals. The ‘- -sex-chr-missing’ option can be used to set problematic genotypes to missing as follows: Y chromosome genotypes for non-male individuals (sex=F or sex=U in the .ind file) since females should not carry Y chromosomes, and heterozygous X chromosome SNPs for male (sex=M in .ind file) since males have only one X chromosome copy.

*Data transformation* optionally performs random pseudo-haploidisation (‘- -random-haploidise’) to convert heterozygous genotypes to homozygous states at random, producing pseudo-haploid data which is the standard in the analysis of low-coverage ancient DNA data. Allele polarization (‘- -polarize’) flips alleles to ancestral/derived orientation using two different approaches: (i) file-based polarization using a two-column input file containing of SNP IDs and their corresponding ancestral allele, or (ii) sample-based polarization using a specific individual from the dataset as an ancestral reference. Polarization excludes SNPs with missing or heterozygous in the reference individual.

*The statistics and output generation* step computes per-SNP allele frequencies and missingness across the dataset (‘- -freq’) and per-individual missing genotype rates (‘- -missing’), providing essential metrics for data quality control and filtering decisions.

While individual-level missing rate filtering is not available as a single flag, it can be performed in two steps: first compute per-individual missingness, then subset individuals as described above based on derived thresholds. Despite involving two steps, this workflow remains fast and does not require data conversion.

When ‘- -verbose’ is enabled, *pygenstrat* writes the logs of removed SNPs (.removed.snp) with specific reasons.

*Processing order: pygenstrat* follows a processing sequence similar to *PLINK* (Chang *et al.* 2015), applying first subsetting and then the calculations. The complete workflow proceeds as follows (i) metadata updates (‘- -update-ind’, ‘- -update-snp’, ‘- -genetic-map’, ‘- -flip-strand’) applied to input files, (ii) allele polarization (‘- -polarize’) and (iii) random pseudo-haploidisation (‘- -random-haploidise’), (iv) individual and/or population filtering (‘- -keep-indv’, ‘- -remove-indv’, ‘- -keep-pop’, ‘- -remove-pop’) applied globally and then for each chunk, (v) sex chromosome handling (‘- -sex-chr-missing’, ‘- -ignore-sex’, ‘- -ignore-unknown’), (vi) SNP ID-based selection (‘- -keep-snps’ or ‘- -remove-snps’) with pre-computed mask applied per chunk, (vii) genomic region or chromosome filtering (‘- -keep-region’, ‘- -remove-region’, ‘- -keep-chr’, ‘- -remove-chr’), (viii) statistics calculations (‘- -missing’ and ‘- -freq’), and finally (ix) genotype missing-rate filtering (‘- -geno’), and/or (ix) minor allele frequency filtering (‘- -min-maf’ and ‘- -max-maf’).

While primarily used as a command-line tool, the modular architecture of *pygenstrat* enables its use as a library within Python scripts. For example, the EigenstratReader() class provides chunk-based iteration over genotype data, enabling memory-efficient computation of population genetic statistics such as f-statistics without loading entire datasets into memory. Additional functions such as filter_by_maf(), and calculate_snp_stats() can be imported to build specialized analysis pipelines that integrate with existing Python-based population genetic tools/scripts.

## 3 Results

The performance of *pygenstrat* and *convertf* was systematically compared using the largest available human ancient DNA dataset, the Allen Ancient DNA Resource AADR v62.0 (Mallick *et al*. 2024, Mallick and Reich 2024), downloaded in April 2025, comprising 1 233 013 SNPs (1 150 639 autosomal, 49 704 X chromosome and 32 670 Y chromosome SNPs) and 17 629 individuals. This dataset represents one of the largest publicly available ancient DNA compilations (up to September 2025) and provides a realistic benchmark for typical workflows.

Benchmarks were tested for common operations: format conversion (ANCESTRYMAP to EIGENSTRAT and vice versa), individual filtering (subsetting to 25%, 50%, and 75% of samples), SNP filtering (subsetting to 25%, 50% and 75% of SNPs), genomic region filtering [e.g. using the 1000 Genomes accessibility mask (Auton *et al*. 2015)]. All these operations were tested for both software and performed on a Linux/Ubuntu server with 40 cores (80 threads) and 755 GB memory using a single thread. *convertf* version 8600 implemented in ADMIXTOOLS (version 8.0.2) was used and *pygenstrat* (version 1.0) runs were executed with default chunk size (1000 SNPs). Wall-clock time and resident set size (RSS) were measured using the shell time utility for consistency.

*pygenstrat* demonstrated consistent 2×–15× speedups across all operations compared to *convertf*. Format conversion showed the largest improvement (15× faster), while combined filtering operations averaged 8x speedups. Memory usage remained stable at < 0.5 GB RAM regardless of dataset size due to chunked processing, compared to *convertf’*s ∼6 GB peak memory usage that scales with input size. Run times and memory usages for additional features of *pygenstrat* that are not included (e.g. MAF filtering) or not functional in *convertf* (e.g. remove SNPs or individuals based on their missing rates) were also evaluated (see Table 1, available as supplementary data at *Bioinformatics Advances* online).

*pygenstrat* outputs are fully compatible with ADMIXTOOLS and EIGENSOFT workflows. The only exception is the custom hash validation system used by *convertf*, which adds a unique hash to ANCESTRYMAP files to verify the file during downstream analysis. This custom hash system could not be replicated in *pygenstrat* due to its undocumented implementation. Therefore, users should set ‘hashcheck: NO’ in ADMIXTOOLS/EIGENSOFT parameter files when processing *pygenstrat*-generated ANCESTRYMAP files. Additionally, currently *pygenstrat* does not support the newer EIGENSTRAT TGENO format, the binary format similar to ANCESTRYMAP but with the genotype matrix transposed (individual-by-SNP rather than SNP-by-individual).

## 4 Discussion

The main advantages of *pygenstrat* can be summarized as follows. First, it addresses critical computational bottlenecks in ancient DNA research by providing efficient, feature-rich EIGENSTRAT processing capabilities. Its 2×–15× performance improvements over *convertf* directly accelerate all downstream analyses, from initial data quality control through complex population genetic modelling. Second, the sequential filtering approach implemented in *pygenstrat* prevents analytical errors, such as calculating allele frequencies including individuals that would later be removed. Third, the extended filtering capabilities eliminate the need for multi-step processing workflows, which reduces overall processing time and minimizes the risk of user error.

The Python implementation facilitates integration with the growing ecosystem of Python-based population genetic tools and enables rapid development of custom analysis pipelines. As ancient DNA datasets continue growing in scale and complexity, *pygenstrat* provides a robust foundation for future methodological developments.

## Supplementary Material

vbag022_Supplementary_Data

## Data Availability

The data underlying this article are available from the Allen Ancient DNA Resource (AADR) at https://doi.org/10.7910/DVN/FFIDCW and were used in this paper for benchmarking. The datasets are derived from sources in the public domain.

## References

[vbag022-B1] Auton A , Brooks LD, Durbin RM et al; 1000 Genomes Project Consortium. A global reference for human genetic variation. Nature 2015;526:68–74. 10.1038/nature15393 26432245 PMC4750478

[vbag022-B2] Chang CC , Chow CC, Tellier LC et al Second-generation PLINK: rising to the challenge of larger and richer datasets. GigaScience 2015;4:7. 25722852 10.1186/s13742-015-0047-8 PMC4342193

[vbag022-B3] Lamnidis TC. EigenStratDatabaseTools: a set of tools to compare and manipulate the contents of EingenStrat databases, and to calculate SNP coverage statistics in such databases. GitHub, 2022. https://github.com/TCLamnidis/EigenStratDatabaseTools (30 November 2025, date last accessed).

[vbag022-B4] Mallick S , Micco A, Mah M et al The Allen Ancient DNA Resource (AADR) a curated compendium of ancient human genomes. Sci Data 2024;11:182. 10.7910/DVN/FFIDCW 38341426 PMC10858950

[vbag022-B5] Patterson N , Moorjani P, Luo Y et al Ancient admixture in human history. Genetics 2012;192:1065–93. 22960212 10.1534/genetics.112.145037 PMC3522152

[vbag022-B6] Patterson N , Price AL, Reich D. Population structure and eigenanalysis. PLOS Genet 2006;2:e190. 17194218 10.1371/journal.pgen.0020190 PMC1713260

[vbag022-B7] Schmid C , Ghalichi A, Lamnidis TC et al Poseidon—a framework for archaeogenetic human genotype data management. Elife 2024;13:RP98317. 10.7554/eLife.98317.1

